# Influence of a Dispersant on the Types and Growth of Microbial Hydrocarbon Degraders in a Crude Oil-contaminated Medium

**DOI:** 10.5696/2156-9614-7.14.62

**Published:** 2017-06-22

**Authors:** Temitope O. Sogbanmu, Victoria F. Doherty, Olanike M. Buraimoh, Olawumi F. Arokoyu

**Affiliations:** 1 Ecotoxicology and Conservation Unit, Department of Zoology, Faculty of Science, University of Lagos, Lagos, Nigeria; 2 Environmental Biology Unit, Department of Biological Sciences, Faculty of Science, Yaba College of Technology, Lagos, Nigeria; 3 Department of Microbiology, Faculty of Science, University of Lagos, Lagos, Nigeria

**Keywords:** crude oil, dispersant, hydrocarbon-degrading bacteria, hydrocarbon-degrading fungi, biodegradation, water

## Abstract

**Background:**

Dispersants are first order response strategies for oil spill cleanup in an aquatic environment. However, their effects on the biodegradation capacity of indigenous hydrocarbon-degrading microorganisms are little known.

**Objectives:**

The influence of a dispersant (DS/TT/066) on the type(s) and growth of hydrocarbon-degrading bacteria (HDB) and hydrocarbon-degrading fungi (HDF) in a crude oil-contaminated medium (water) was investigated in the laboratory for 28 days.

**Methods:**

The experiment was set up in duplicates with the first set containing Forcados light crude oil (FLCO) alone in water while the other was a mixture of FLCO and DS/TT/066 (ratio 9:1 v/v). Identification and enumeration of HDB and HDF were conducted according to standard methods. Total petroleum hydrocarbons (TPH) in the test media was analyzed using a gas chromatography/flame ionization detector.

**Results:**

The results showed that HDB identified in the FLCO alone included Pseudomonas aeruginosa (day 0), Proteus vulgaris (day 14), P. aeruginosa and Kliebsiella pneumoniae (day 28). However, in the mixture, Escherichia coli was identified on day 14 in addition to the other species observed in FLCO alone. HDF identified in FLCO alone were Candida krusei and Candida albicans (days 0 and 14), Trichosporon cutaneum and C. albicans (day 28). In the mixture, HDF identified were C. albicans (day 0), C. albicans and Aspergillus spp. (days 14 and 28)″ Furthermore, the mixture enhanced the growth of HDBF (average counts: 32.5 × 10^7^ and 225 × 10^6^ cfu/mL) compared to FLCO alone (17.5 × 10^7^ and 17.5 × 10^6^ cfu/mL) by day 14 respectively. Total petroleum hydrocarbon reduction was highest (85%) in the mixture compared to 5% in FLCO alone by day 14.

**Conclusions:**

The study demonstrated the biodegradation efficiency of E. coli, P. vulgaris (bacteria), C. albicans and Aspergillus spp. (fungi) in a crude oil-contaminated aquatic environment in conjunction with dispersant use. Further studies in the field are recommended in order to explore their potential for rapid and large scale crude oil clean-up operations.

## Introduction

In the aquatic environment, crude oil spills have widespread deleterious effects and require the deployment of various control and clean-up strategies including the use of dispersants. Dispersants are chemicals that are applied to oil to break it up into tiny droplets and enhance biodegradation of bulk crude oil by increasing the bioavailable fraction of the hydrocarbon through mobilization of absorbed hydrocarbons or by increasing its effectiveness in aqueous solubility.^1^ Chemical dispersants can be very effective tools in the management of crude oil spilled in aquatic environments.^2^ However, several important factors must be considered in deciding whether to use dispersants, including their physical effectiveness under conditions of expected use, the effect of dispersants on the fate of spilled petroleum hydrocarbon products, and the type of joint action toxicity exhibited by the mixtures.^3–6^ Dispersant effectiveness has been defined as the amount of oil that the dispersant puts into the water column compared to the amount of oil that remains on the surface.^7^

Microbial biodegradation assays of pollutants have intensified in recent years as efforts have been made to find sustainable ways to cleanup contaminated environments.^8^ Biodegradation of petroleum hydrocarbon (crude oil) is a complex process that depends on the nature and amount of the hydrocarbons present.^9^ Microbial degradation is the major and ultimate natural mechanism by which crude oil is cleaned up in the environment.^10^ The mechanism for degradation of crude oil hydrocarbons has been studied extensively, and a number of microorganisms, including bacteria and fungi, have been isolated and characterized for their ability to degrade crude oil hydrocarbons.^11–13^ Bacteria are the most active agents in petroleum degradation and work as primary degraders of spilled oil in the environment. In addition, several bacteria are known to feed exclusively on hydrocarbons.^14^ Some fungi are also capable of utilizing crude oil.^15^ Typical bacteria groups previously identified for their degrading capacity include Pseudomonas spp., Gordonia spp. and Cellulomonas spp., while fungi genera include Aspergillus spp., Penicillium spp., Fusarium spp. and Candida spp.^16^,^17^

According to Mulyono et al., dispersants tend to increase oil biodegradation by increasing the surface area for microbial attack and encouraging migration of the droplets through the water column making oxygen and nutrients more readily available.^18^ Venosa and Holder also reported that degradation was much more rapid for dispersed oil than for non-dispersed oil, because in the non-dispersed control, the microbial culture first had to generate its own biosurfactant to emulsify the oil before substantial degradation could occur.^19^ In addition, Sogbanmu and Otitoloju reported that some dispersants used in Nigeria were capable of enhancing the microbial biodegradation of crude oil.^20^ However, few studies have identified the types of microbial hydrocarbon degraders and the extent of degradation of crude oil in concert with dispersant use in an aquatic environment.

This research consequently aimed to investigate the influence of a named dispersant on the types and growth of hydrocarbon-degrading bacteria and fungi in a crude oilcontaminated medium (water) with a concomitant evaluation of the extent of biodegradation through measurement of leftover total petroleum hydrocarbons over a period of 28 days.

## Methods

### Materials

***Crude Oil:*** Forcados light crude oil (FLCO) used for this experiment was obtained from the Shell production platform in Forcados, Burutu Local Government Area of Delta State, Nigeria. The physico-chemical properties of the crude oil include:

sulphur content = 0.2%, American Petroleum Institute gravity = 60/60 F, rapid vapor pressure = 2.5 psi, and pour point = 25.

It was stored in a sealed plastic vessel in the laboratory at room temperature and used within a period of 30 days.

***Dispersant (DS/TT/066)***: Brown-colored liquid containing surfactant mixed with a hydrocarbon solvent. Manufactured by Oil Pollution Environmental Control Limited, West Yorkshire, Great Britain.

### Test Media Preparation

Mixtures containing FLCO alone and a binary mixture of FLCO:DS/TT/066 (ratio 9:1 v/v) were prepared and made up to 5 mL/L. For the FLCO:dispersant mixture, the proportion of each constituent compound dictated by the ratio was computed and measured out. FLCO was applied to the substrate (water) before transferring the dispersant at the desired concentration into the test container. There were two replicates per mixture and these were exposed over a period of 28 days.

**Abbreviations**
DS/TT/066: Dispersant
*FLCO*: Forcados light crude oil
*HDB*: Hydrocarbon-degrading bacteria
*HDF*: Hydrocarbon-degrading fungi
*TPH*: Total petroleum hydrocarbons

### Isolation and Identification of Hydrocarbon-Degrading Bacteria and Fungi

#### Isolation of Hydrocarbon Degrading Bacteria

Isolation of hydrocarbon degrading bacteria (HDB) was conducted as described by Sogbanmu and Otitoloju.^20^ Briefly, diluent (distilled water) for serial dilution was prepared by pipetting 9 mL into test tubes. They were covered with aluminum foil-wrapped cotton wool and autoclaved at 121°C for 15 minutes. A mineral salts medium (100 mL) to which was added an appropriate quantity of agar-agar (solidifying agent) was brought to boil in a water bath and autoclaved at 121°C for 15 minutes. This was allowed to cool to about 45°C, then aseptically poured into sterile petri dishes and allowed to set. The mineral salts medium used for this study was adapted from Adebusoye et al. with slight modification.^9^ It contained potassium nitrate (1.0 g), epsomite (1.0 g), calcium chloride hexahydrate (0.1 g), iron (II) sulfate (0.05 g), trace elements solution (250 mL), phosphate buffer (1 M, pH 6.8, 20 mL) and distilled water (980 mL) as described by Mittal and Singh.^21^

For isolation, 1 mL of sample mixture was aseptically transferred into a test tube containing 9 mL of distilled water (after cooling) using a micropipette. Ten-fold serial dilution was done up to 7 times (10^7^) and then 0.1 mL of sample (7^th^ dilution) was inoculated onto sterile plates containing cooled agar. The sample was spread on the surface with the aid of a sterile glass spreader for even distribution of colonies or cells. A sterile filter paper to which crude oil had been added and spread evenly was placed on the inside top cover of the petri dish before covering. These plates were stored in an incubator at room temperature for 3 days (optimal growth time for hydrocarbon degrading bacteria).

#### Isolation of Hydrocarbon-Degrading Fungi

Briefly, 9 mL of distilled water was pipetted into a test tube and the tubes were covered with aluminum foil wrapped cotton wool and autoclaved at 121°C for 15 minutes and allowed to cool. Potato dextrose agar (Lab M) was prepared according to the manufacturer's instructions and then autoclaved at 121°C for 15 minutes. The agar was allowed to cool and then poured into sterile petri dishes. For isolation, 1 mL of sample mixtures was taken with the aid of a micropipette and transferred into a test tube containing 9 mL of sterile distilled water (after cooling). Ten-fold serial dilution was done up to 6 times (10^−6^) and then 0.1 mL of sample (6^th^ dilution) was inoculated onto sterile plates containing cooled agar. The sample was spread on the agar surface with the aid of a sterile glass spreader for even distribution of colonies. Incubation was carried out at 28°C for 7 days (optimal growth time for hydrocarbon degrading fungi).

#### Identification of Isolates

The distinct colonies obtained after incubation were sub-cultured onto new (freshly-prepared) nutrient agar plates (bacteria) and new potato dextrose agar plates (fungi) to obtain pure cultures. Pure bacterial and fungal colonies were used for morphological and biochemical characterization to identify the oil degrading microorganisms. Morphological and biochemical tests *(Table 1)* of the bacterial isolates were carried out as previously described by Dubey and Maheshwari.^22^

**Table 1:** Biochemical Test for Identification of Bacteria Isolates

**Test Media**	**Gram staining**	**Cellular morphology**	**Mannitol test**	**Motility test**	**Urease test**	**Citrate test**	**Indole**	**Oxidase**	**Coagulase test**	**Starch hydrolysis**	**Glucose**	**Lactose**	**Gas**	**H2S**	**Maltose**	**Sucrose**	**Xylase**	**Most Probable Identity**
**DAY 0**																		
***FLCO_a_***	−	Rods	+	+	−	+	−	+		−	+	−	−	−	−	−	+	Pseudomonas aeruginosa
***FLCO : D_a_***	−	Rods	+	+	−	+	−	+		−	+	−	−	−	−	−	+	Pseudomonas aeruginosa

**DAY 14**																		
***FLCO_a_***	−	Rods		+	+	−	+	−			+	−	+	+				Proteus vulgaris
***FLCO_b_***	−	Rods		+	+	−	+	−			+	−	+	+				Proteus vulgaris
***FLCO : D_a_***	−	Rods		+	+	−	+	−			+	−	+	+				Proteus vulgaris
***FLCO : D_b_***	−	Rods		+	−	−	−	−			+	+	+	−				Escherichia coli

**DAY 28**																		
***FLCO_a_***	−	Rods	+	+	−	+	−	+		−	+	−	−	−	−	−	+	Pseudomonas aeruginosa
***FLCO_b_***	−	Rods		−	+	+	−	−			+	+	+	−				Klebsiella pneumoniae
***FLCO : D_a_***	−	Rods	+	+	−	+	−	+		−	+	−	−	−	−	−	+	Pseudomonas aeruginosa
***FLCO : D_b_***	−	Rods		−	+	+	−	−			+	+	+	−				Klebsiella pneumoniae

**Abbreviations:** +, indicates presence or positive reaction; −, indicates absence or negative reaction; D, Dispersant; a, Replicate 1; b, Replicate 2

Identification was done with the use of a flow chart based on the manual for the identification of bacteria.^23^ Biochemical tests for identification of fungi isolates are presented in Table 2.

**Table 2:** Biochemical Test for Identification of Fungi Isolates

**Test Media**	**Gram staining**	**Cellular morphology**	**Glucose**	**Galactose**	**Arabinose**	**Maltose**	**Lactose**	**Sucrose**	**Germ tube**	**Most Probable Identity**
**DAY 0**										
***FLCO_a_***	+	oval	+	−	−	−	−	−	−	Candida krusei
***FLCO_b_***	+	oval	+	+	−	+	−	+	+	Candida albicans
***FLCO : D_a_***	+	oval	+	+	−	+	−	+	+	Candida albicans
***FLCO : D_b_***	+	oval	+	+	+	+	−	+	−	Candida albicans
**DAY 14**										
***FLCO_a_***	+	oval	+	+	−	+	−	+	+	Candida albicans
***FLCO_b_***	+	oval	+	−	−	−	−	−	−	Candida krusei
***FLCO : D_a_***	+	oval	+	+	−	+	−	+	+	Candida albicans
***FLCO : D_b_***	+	cylindrical								Aspergillus spp..
**DAY 28**										
***FLCO_a_***	+	oval	−	−	−	−	−	−	−	Trichosporon cutaneum
***FLCO_b_***	+	oval	+	+	+	+	−	+	−	Candida albicans
***FLCO : D_a_***	+	cylindrical								Aspergillus spp..
***FLCO : D_b_***	+	oval	+	+	−	+	−	+	+	Candida albicans

Abbreviations: +, indicates presence or positive reaction; −, indicates absence or negative reaction; D, Dispersant; a, Replicate 1; b, Replicate 2

### Enumeration of Hydrocarbon-Degrading Bacteria and Fungi

The cultured samples of bacteria and fungi which were incubated at room temperature for 3 days and 28°C for 7 days, respectively, were brought out and colonies in the different petri dishes were observed, counted and recorded.^20^

### Collection, Extraction and Analysis of Total Petroleum Hydrocarbon in Water Samples

With the aid of a micropipette, 100 mL of water sample was obtained and transferred into a 120 mL bottle. The extraction method for the analysis of total petroleum hydrocarbons (TPH) in the water samples was according to the modified methods of the International American Section of the International Association for Testing Materials.^24^,^25^ Then 100 ml of the water sample was transferred into a 1 L separatory funnel and 30 mL of the redistilled hexane and dichloromethane in ratio 3:1 was added. The separatory funnel was shaken vigorously for about 20 mins with periodic venting to release vapor pressure. The organic layer was allowed to separate for 10 minutes and was recovered into a 250 mL flask. The aqueous layer was re-extracted twice with 30 ml of the extractant. The combined extract was dried by passing through the funnel containing the anhydrous sodium sulphate. The dried extract was concentrated with a stream of nitrogen gas. The mixture was concentrated to 1 mL before the gas chromatography analysis. All quality assurance/quality control requirements were ensured. A series contained a blank, a standard of known concentration and samples. Daily calibrations were performed. The R^2^ value for the calibration curve was 0.9996–8. Gas chromatography (Hewlett Packard gas chromatograph 6890 coupled to a flame ionization detector) conditions were as follows: injection type (split injection), split ratio (20:1), carrier gas (nitrogen), inlet temperature (250°C), column type, (HP 5), column dimensions (30 m × 0.25 mm × 0.25 μm), and oven program: initial temperature at 60°C, and first ramping at 10°C/min for 20 mins, maintained for 2 mins. The second ramping occurred at 15°C/min for 4 min, maintained for 4 min, detector (flame ionization detector), detector temperature (320°C), hydrogen pressure (28 psi), and compressed air (40 psi). Quantitative analysis was carried out with an HP 6890 powered with HP Chemstation Rev. A09.01(1206) software and utilized automated integration to obtain total area counts of chromatogram peaks. The data for total petroleum hydrocarbon concentration measured in the samples by gas chromatography/flame ionization detector were expressed in mg/L.

### Data Analysis

The results of the analysis of total hydrocarbon degrading bacteria and hydrocarbon degrading fungi were expressed in colony forming units per milliliter (cfu/mL) and represent average counts of colonies. Microsoft Excel version 2010 was used to obtain the mean and standard deviation of the data as well as plot the graphs.

## Results

### Identification of Hydrocarbon-Degrading Bacteria and Fungi in the Test Media

The results of the HDB showed that FLCO alone supported the growth of Pseudomonas aeruginosa at day 0, Proteus vulgaris at day 14, and P. aeruginosa and Kliebsiella pnuemoniae by day 28. The FLCO:DS/TT/066 mixture also supported the growth of the same bacteria species as FLCO on days 0 and 28. However, for day 14, in addition to P. vulgaris, Escherichia coli was identified *(Tables 1 and 3).*

**Table 3:** Summary of Results Showing Hydrocarbon-Degrading Bacteria and Fungi Identified in Test Media

**Test Media**	**Day 0**	**Day 14**	**Day 28**
**Hydrocarbon-Degrading Bacteria**			
**FLCO alone**	Pseudomonas aeruginosa	Proteus vulgaris	Pseudomonas spp. *&* Klebsiella spp.
**FLCO:DS/TT/066**	Pseudomonas aeruginosa	Proteus vulgaris *&* Escherichia coli	Pseudomonas spp. *&* Klebsiella spp.
**Hydrocarbon-Degrading Fungi**			
**FLCO alone**	Candida krusei	Candida albicans	Trichosporon cutaneum *&* Candida albicans
**FLCO:DS/TT/066**	Candida albicans	Candida albicans *&* Aspergillus spp.	Candida albicans & Aspergillus spp.

**Abbreviation:** D, Dispersant

The HDF identified in the medium containing FLCO alone was Candida krusei and Candida albicans at day 0, Candida albicans at day 14, Trichosporon cutaneum and Candida albicans at day 28. The FLCO:DS/TT/066 mixture favored the growth of C. albicans alone at day 0, C. albicans and Aspergillus spp. at days 14 and 28 *(Tables 2 and 3).*

### Enumeration of Hydrocarbon-Degrading Bacteria and Fungi in the Test Media

**Figure:**
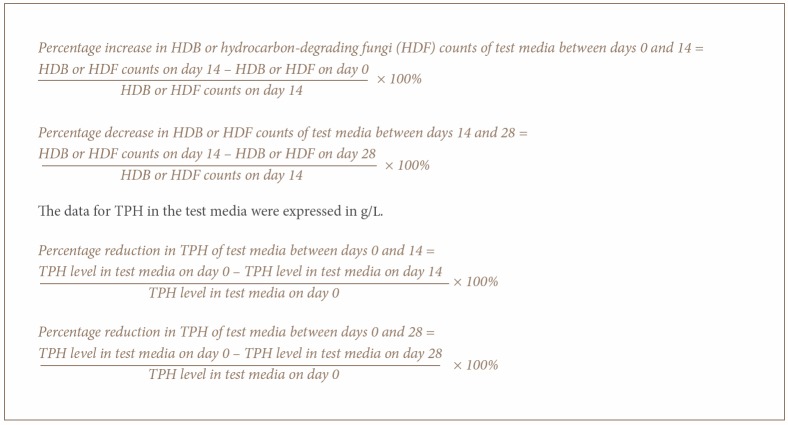


**Figure 1:**
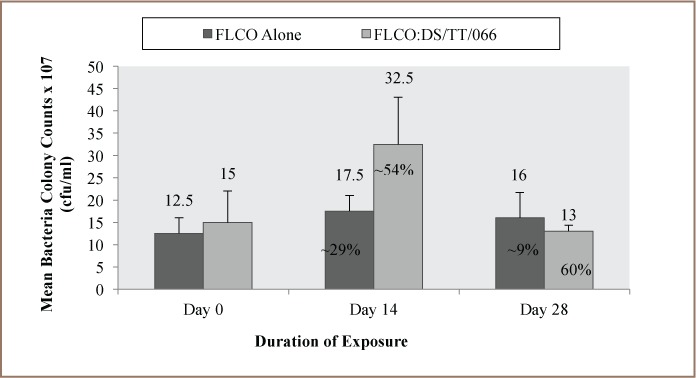
Mean bacteria colony counts (cfu/mL) in test media over a period of 28 days (n=2)

**Figure 2:**
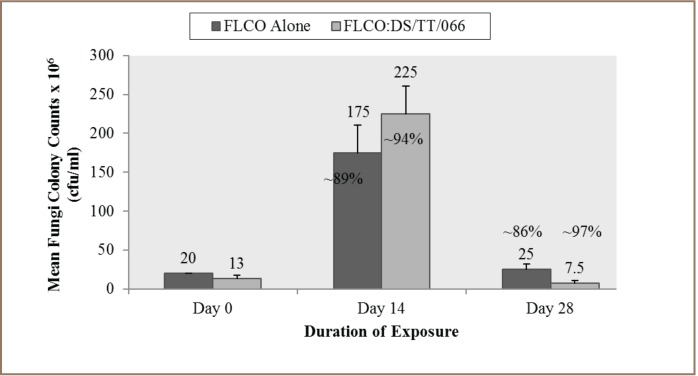
Mean fungi colony counts (cfu/mL) in test media over a period of 28 days (n=2)

The enumeration of HDB and HDF in the test media revealed that the medium containing FLCO alone had mean HDB counts of 12.5, 17.5, 16.0 × 10^7^ cfu/mL and HDF counts of 20.0, 17.5, 25.0 × 10^6^ cfu/mL at days 0, 14 and 28 respectively. On the other hand, the FLCO:DS/TT/066 mixture had mean HDB counts of 15.0, 32.5, 13.0 ×10^7^ cfu/mL and HDF counts of 13.0, 225.0, 7.5 × 10^6^ cfu/mL at days 0, 14 and 28 respectively *(Figure 1 and 2).*

Comparison of the percentage increase in HDB from day 0 to day 14 in the test media showed a ~29% and ~54% increase for FLCO alone and the FLCO:DS/TTT/066 mixture, respectively. A decrease of 9% and ~60%, respectively, was observed in the test media from day 14 to day 28 *(Figure 1).* For HDF, the percentage increase from day 0 to day 14 in the test media was ~89% and ~94% for FLCO alone and FLCO:DS/TTT/066 mixture, respectively. A decrease of ~86% and ~97%, respectively, was observed in the test media from day 14 to day 28. Hence, in both media, the highest HDB and HDF was observed on day 14 *(Figure 2).*

### Total Petroleum Hydrocarbons Analysis

**Figure 3:**
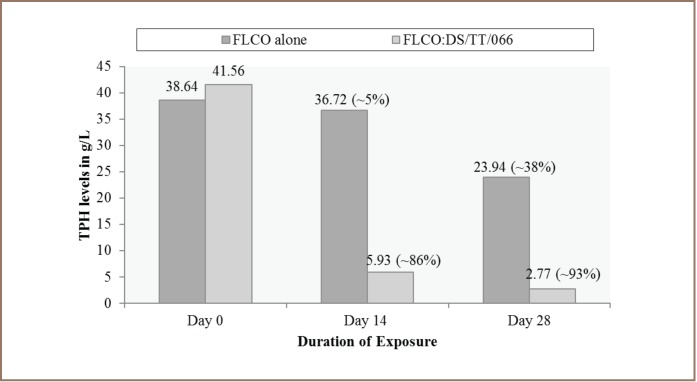
Total petroleum hydrocarbons levels in test media over a period of 28 days

The results of the analyses of TPH in the test media revealed that the FLCO:DS/TT/066 mixture had a higher reduction in the TPH at days 14 and 28 (86% and 93%, respectively) compared to the mixture containing FLCO alone (5% and 38% respectively). Consequently, the highest reduction in TPH levels was achieved in the FLCO:DS/TT/066 mixture at day 28 compared to FLCO alone *(Figure 3).*

## Discussion

The identified HDB in the test media showed the types of bacteria that were capable of utilizing the crude oil as a carbon source and thus degrading it. These microorganisms have also been reported in other studies to be capable of hydrocarbon degradation.^26^ However, in addition to the identified bacteria in both test media, E. coli was also identified in the dispersed crude oil mixture on day 14 compared to the undispersed oil. This suggests that E. coli was able to degrade the oil in the presence of a dispersant. The mechanism of hydrocarbon degradation in bacteria has been studied and shown to involve the production of biosurfactant which promotes the cracking of hydrocarbon molecules by micelle formation, increasing their mobility, bioavailability and exposure to bacteria, thus favoring hydrocarbon biodegradation.^27^ The presence of the dispersant seems to have hastened the process of biosurfactant production so that the bacteria were able to act faster than in the undispersed medium.

In addition, the presence of E. coli in the dispersed mixture and not in the undispersed mixture could also mean that some components of the crude oil which E. coli specializes in degrading were available in the dispersed medium, but not the undispersed medium. Other bacteria (Pseudomonas aeruginosa, Proteus vulgaris and Klebsiella pneumoniae) identified in both media shows that these species are broad spectrum and hence can act both in the presence and absence of a dispersant. P. aeruginosa has been used for bioremediation of places contaminated by petroleum.^28^ In addition, the different bacteria identified showed some level of specificity in the timing of occurrence. P. aeruginosa were only identified on day 0 and 28, which suggests that they were only available to act on components of the oil that were present in the beginning and towards the end of the degradation process. It was also noted that P. vulgaris was only identified in both test media on day 14. This suggests that this bacterium specializes in the degradation of components of oil that would have been available about two weeks into the degradation process.

For the HDF, the two test media showed slightly different fungi makeup. Only Candida albicans appeared to cut across both test media and throughout the test duration. This could mean that the fungus has the capability to degrade various components of the oil at various stages of degradation. It could thus be said that the fungus is broad spectrum. Aspergillus spp. was identified in the dispersed crude oil only on days 14 and 28. This suggests that the fungus is able to act in the presence of a dispersant since it was not identified in the undispersed oil. On the other hand, Candida krusei identified on days 0 and 14 and Trichosporon cutaneum identified on day 28 in the undispersed oil suggests that these fungi could not act in the presence of a dispersant since they were not identified in the dispersed medium.

The gradual increase in HDB and HDF observed in both test media up to day 14 followed by a decrease from days 14 to 28 suggests the degradation of various components of the crude oil by different bacteria and fungi. This finding is in agreement with a study by Sogbanmu and Otitoloju who reported a similar response of HDB evaluated in dispersed oil mixtures and undispersed oil.^20^ However, the FLCO:DS/TT/066 mixture revealed a higher incidence of HDB and HDF counts by day 14 compared to FLCO alone. This could be attributed to the fact that the dispersant broke up the crude oil emulsion increasing the surface area of the oil thus making more bioavailable fractions for the microorganisms to act upon. However, the dispersed crude oil showed a marked decrease in microbial counts between days 14 and 28 compared to the undispersed oil considering the percentages. This suggests that the various components of the crude oil had been utilized by the microorganisms. Hence, unavailability of substrates to act upon resulted in the decrease in the microbial counts. Sim and Ward also reported that commercial chemical surfactants may also be used to boost microbial degradation of hydrocarbons, although different types of surfactants would have different effects.^29^

In order to certify that actual biodegradation had occurred, total petroleum hydrocarbons in the test media was measured. The results showed a reduction of TPH in both media from days 0 to 28. However, the dispersed oil medium showed a very sharp decrease in TPH by day 28 in particular, compared to the undispersed oil. These results suggest that the HDB and HDF in the media, especially those identified on day 14, did the greater work of degradation as the percentage reduction in TPH between day 0 and 14 was several folds higher than that observed between days 14 and 28 in the dispersed oil medium. Also, the level of TPH left over in the dispersed oil medium after 28 days revealed that biodegradation was several times faster in the presence of the dispersant. The results of the TPH levels correlate with the results of the HDB and HDF counts in the dispersed oil medium. The level of TPH in the dispersed oil medium was higher on day 0 which suggests that the crude oil was more available in the medium, thus stimulating the HDB and HDF growth as seen in the counts on day 14. This is in accordance with the report by Souza et al., which argued that the larger the degrading microorganism population, the quicker and more efficient the bioremediation process.^27^ The increase in HDB and HDF counts means that the microorganisms were actively degrading the TPH which resulted in a very steep reduction in TPH by day 14. Suwansukho et al. noted that biosurfactants are usually produced in the exponential or stationary phase of microbial growth when there is a high cellular density.^30^ These biosurfactants synthesized as metallic products of different microorganisms degrade hydrocarbons and use them as a carbon source.^31^,^32^ However, the gradual depreciation in TPH in the dispersed oil medium resulted in the reduction in HDB and HDF counts between days 14 and 28. This was shown in the slight decrease in TPH between days 14 and 28 compared to days 0 and 14.

## Conclusions

The diversity of microorganisms capable of biodegrading pollutants such as oil is vast and yet to be fully exploited. In addition, with advances in research, the number of identified species grows every year.^33^ The present study identified the types of HDB and HDF in a dispersed oil medium, with a focus on the identification of specific bacteria and fungi in the dispersed medium as opposed to the undispersed medium. The efficacy of degradation seen in the dispersed oil medium suggests that these specific bacteria and fungi might possess greater biodegradation potentials which have yet to be explored in the presence of a dispersant. Therefore, we recommend that further studies, both laboratory and field based, be carried out to take advantage of the biodegradation potentials of these naturally occurring microorganisms in tandem with responsible dispersant use to combat crude oil pollution in aquatic ecosystems. In particular, we recommend further research to better understand the specific roles of the identified HDB and HDF colonies in the degradation of crude oil in concert with dispersant use under natural conditions.
